# Phosphoproteomic analysis of the response to DNA damage in *Trypanosoma brucei*

**DOI:** 10.1016/j.jbc.2024.107657

**Published:** 2024-08-14

**Authors:** Emilia McLaughlin, Monica Gabriela Zavala Martinez, Annick Dujeancourt-Henry, Thibault Chaze, Quentin Giai Gianetto, Mariette Matondo, Michael D. Urbaniak, Lucy Glover

**Affiliations:** 1 Institut Pasteur, Université Paris Cité, Trypanosome Molecular Biology, Department of Parasites and Insect Vectors, Paris, France; 2 Sorbonne Université, Collège doctoral, Paris, France; 3 Institut Pasteur, Université Paris Cité, Proteomics Platform, Mass Spectrometry for Biology Unit, Centre National de la Recherche Scientifique, UAR 2024, Paris, France; 4 Institut Pasteur, Université Paris Cité, Bioinformatics and Biostatistics HUB, Paris, France; 5 Division of Biomedical and Life Sciences, Faculty of Health and Medicine, Lancaster University, Lancaster, UK

**Keywords:** phosphoproteomics, *Trypanosoma brucei*, DNA damage response, DNA break

## Abstract

Damage to the genetic material of the cell poses a universal threat to all forms of life. The DNA damage response is a coordinated cellular response to a DNA break, key to which is the phosphorylation signaling cascade. Identifying which proteins are phosphorylated is therefore crucial to understanding the mechanisms that underlie it. We have used stable isotopic labeling of amino acids in cell culture-based quantitative phosphoproteomics to profile changes in phosphorylation site abundance following double stranded DNA breaks, at two distinct loci in the genome of the single cell eukaryote *Trypanosoma brucei.* Here, we report on the *T. brucei* phosphoproteome following a single double-strand break at either a chromosome internal or subtelomeric locus, specifically the bloodstream form expression site. We detected >6500 phosphorylation sites, of which 211 form a core set of double-strand break responsive phosphorylation sites. Along with phosphorylation of canonical DNA damage factors, we have identified two novel phosphorylation events on histone H2A and found that in response to a chromosome internal break, proteins are predominantly phosphorylated, while a greater proportion of proteins dephosphorylated following a DNA break at a subtelomeric bloodstream form expression site. Our data represent the first DNA damage phosphoproteome and provides novel insights into repair at distinct chromosomal contexts in *T. brucei.*

One of the most toxic lesions to the genome is a DNA double-strand break (DSB), where breaks occur simultaneously in the phosphate backbone of two complementary DNA strands. DSBs in the DNA can arise due to endogenous processes in the cell, such as replication fork collapse or stalling, and can also result from exogenous agents, such as chemicals or ionizing radiation (1). DSB repair (DSBR) is a coordinated program of events initiated by a signaling cascade, with protein phosphorylation at its core. In eukaryotes the DSBR is a conserved process initiated by detection and processing by the MRE11, RAD50, and NBS1 (MRN) complex and leads to the recruitment of two key kinases, ataxia-telangiectasia mutated (ATM) and ATM and Rad3-related kinase (ATR), which are the master regulators of the DNA damage response (DDR) (2). Subsequently, a phosphorylation cascade is initiated with some 900 substrates modified (3). One of the key substrates of ATM is S139 of the histone variant H2AX, which when phosphorylated is termed γH2AX and is regarded as an early marker of repair in mammals (4, 5). γH2AX spreads along large regions of chromatin fiber bidirectionally from the break site (3) which aids the recruitment of chromatin remodeling factors, allows DNA damage proteins to access the break (6) and concentrates repair factors at the damaged site (7).

Understanding phosphorylation cascades have been driven by stable isotopic labeling of amino acids in cell culture (SILAC) (8) based quantitative phosphoproteomics (3, 9, 10, 11). In human cells, SILAC phosphoproteomics has identified over 900 phosphorylation sites associated with ionizing radiation induced DNA damage, revealing a series of interconnected networks in the DDR including proteins associated with DNA repair, replication, and chromatin modifications (3). Over 70% of the phosphorylation sites identified are targets of the ATM kinase, and many of these substrates are themselves kinases, highlighting the central role of the phosphorylation cascade in the DDR.

Human African trypanosomiasis is a fatal vector borne disease caused by the protozoan parasite *Trypanosoma brucei.* In the mammalian host the parasite is found in the bloodstream, adipose tissue (12) and skin (13, 14). Here, the parasite is exposed to attack by the host immune system and is protected by a dense variant surface glycoprotein (VSG) coat, which is periodically exchanged by antigenic variation (15, 16). *VSGs* are exclusively expressed from a subtelomeric bloodstream form expression sites (BESs) (17), of which there are approximately 15, with only one active (the active BES) and the rest silenced. The majority of *VSG* genes are located in arrays in the subtelomeric regions of the megabase chromosomes, and also occasionally at chromosome internal regions (18) and act as a repertoire for antigenic variation. There has been much debate into what drives antigenic variation, with DSBs (19, 20), replication-derived fragility from the early replication of the BES (21, 22) or the formation of RNA:DNA hybrids (23, 24) all being implicated. In *T. brucei* repair occurs predominantly *via* homologous recombination (HR) (25). Repair by microhomology-mediated end joining accounts for approximately 5% of repair, but up to 25% at the active BES (20). Antigenic variation occurs mainly by gene conversion events, where the active *VSG* is deleted and replaced by a silent donor (26, 27, 28, 29), but crossover switching events, where two *VSGs* are exchanged, have also been observed (28, 30, 31, 32).

Several DNA damage linked proteins directly influence repair and antigenic variation, and within the homologous repair pathway, sequence diversity among the genes facilitating repair suggests functional divergence within the pathway as well (33). ATR mediates signal transduction in trypanosomes (34) and loss leads to an increase in nuclear DNA damage and VSG switching (35). The RecQ-like helicase is required for genome repair and mutants show elevated VSG switching by telomere recombination and *VSG* gene conversion events (22). RAD51, the primary recombinase in DNA repair, is required for homology searching and DNA strand exchange and is loaded onto single-strand DNA by BRCA2 (36, 37). In Trypanosoma *brucei*, BRCA2 is essential for homologous recombination, DNA replication, cell division, and antigenic variation (38, 39), while RAD51 essential for HR and *rad51* null mutants have impaired, VSG switching (40, 41, 42, 43). In trypanosomes, five RAD51-related proteins, RAD51 to 3, 4, 5, and 6 are important for DSBR, but only RAD51 to 3 contributes to VSG switching (44). Early recognition and processing of a DNA break *via* the MRE11, RAD50, and NBS1 complex is important for both detection and signaling of a DSB. In *Leishmania*, MRE11 maintains genomic integrity and although in *T. brucei* does not affect the rate of VSG switching (40, 45, 46, 47), both MRE11 and RAD50 promote recombination using longer stretches of homology which restricts the diversity of *VSG* genes used for antigenic variation (48). At a chromosomal internal locus, MRE11 is required for efficient resection (48). The RECQ/TOPO3/RMI1 (RTR) complex which includes the RecQ-family helicase, a topoisomerase IIIα, and RMI1/2 suppress these mitotic crossover and removes recombination intermediates (49). In trypanosomes, *VSG* gene conversion and cross over events can be suppressed by the RECQ/TOPO3/RMI1 complex components *Tb*TOPO3α and *Tb*RMI1 or act in concert with RAD51 and RMI1 (42, 50). During the DNA damage repair cycle, the G_2_/M checkpoint prevents division of unrepaired DNA and preserves genome integrity. Although trypanosomes do show cells arrested in G_2_/M following a DSB, some cells do continue to replicate and divide their DNA with a DNA break. This suggests a level of tolerance to DNA damage greater than that seen in other eukaryotes (51, 52), perhaps aiding homology searching for antigenic variation. Despite the importance of HR in evasion of the host immune system only one DNA damage associated phosphorylation site has been identified in *T. brucei,* that of γH2A (53), which is analogous to H2AX in mammalian cells (4). In *T. brucei*, H2A T131 is phosphorylated in response to a DSB, typically during S or G2-phases of the cell cycle.

Here, we use a quantitative single-locus phoshoproteomic approach to characterize changes in phosphorylation site abundance in response to a DSB at two distinct loci (i) a chromosome internal locus where classic HR is the dominate form of repair (25) and (ii) the active BES where repair facilitates antigenic variation (20). We found that there is a striking distinction between the proteins phosphorylated in response to a chromosome internal DSB and one at the active BES and identify two novel DNA damage associated phosphorylation sites on Histone H2A.

## Results

### Adaptation of the ^1^HR and VSG^up^ cell lines to SILAC medium

**Figure 1:**
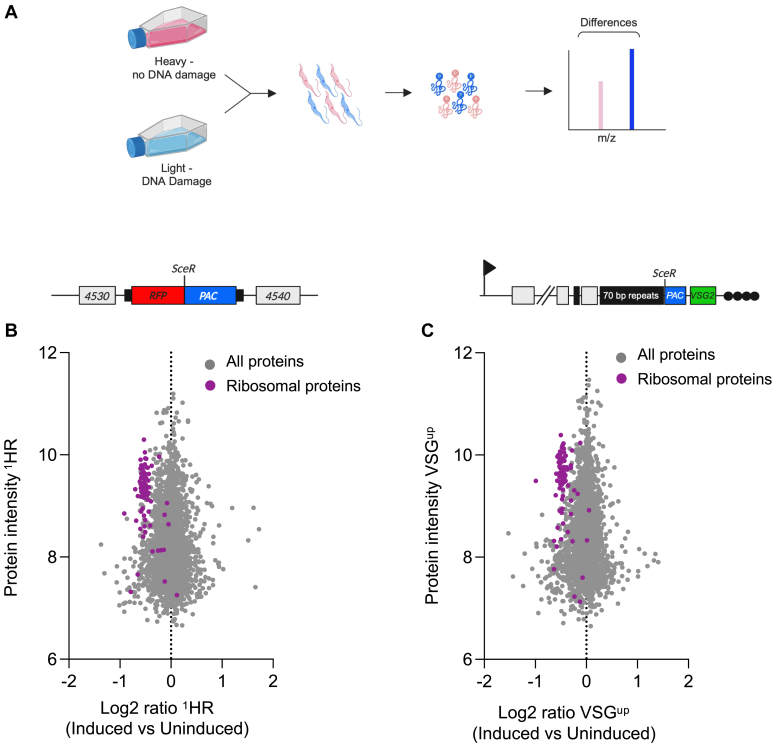
**The**^**1**^**HR and VSG**^**up**^**DSB proteome.***A*, proteomic SILAC strategy for *Trypanosoma brucei* to identify proteins phosphorylated in response to a single double-strand break. *T. brucei* cells were labeled with either heavy or light amino acids and subject to DNA damage. The protein extracts were mixed at a 1:1 ratio and analyzed by mass spectrometry. *B*, *upper panel*: schematic of chromosome 11 Tb927.11.4530/40 locus following modification to generate the ^1^HR chromosome-internal DSB cell line with the I-*SceI* recognition site, SceR, highlighted. The DSB site is flanked upstream by red fluorescent protein (*RFP*) and puromycin-N-acetyltransferase (*PAC*) downstream. The site is positioned at an intergenic region between Tb927.11.4530 and Tb927.11.4540, shown as “4530” and “4540”, respectively. *Black boxes* are tubulin intergenic sequences. *Lower panel*: total proteins identified in ^1^HR. The *x*-axis is the Log_2_ value of the ratio of each protein given as its presence in the DSB induced *versus* uninduced sample. The *y*-axis is the intensity of a given protein in the sample. Ribosomal proteins are highlighted in *purple*, all other proteins shown in *dark gray*. *C*, *upper panel*: schematic showing the VSG^up^ cell line set up showing the modified BES1 on chromosome 6a. An I-*SceI* meganuclease recognition site is inserted upstream of the actively expressed *VSG-2*, shown with a *green box*. The *SceR* is flanked downstream by a puromycin-N-acetyltransferase gene (PAC). *Arrow*; native promoter of the expression site, *white boxes*; genes, *solid black box*; 70 bp repetitive sequence, *black circles*; telomere. *Lower panel*: The VSG^up^ proteome, with details as described in *B*. Ribosomal proteins are highlighted in *purple*, all other proteins shown in *dark gray*. Schematics generated with BioRender. BES, bloodstream form expression site; DSB, double-strand break; HR, homologous recombination; SILAC, stable isotopic labeling of amino acids in cell culture; VSG, variant surface glycoprotein.

SILAC experiments require the metabolic incorporation of stable isotope labeled amino acids present in the cell culture medium. Trypanosomatids are auxotrophic for arginine (R) and lysine (K) (54) and we therefore used cell culture medium lacking in both and supplemented with either the “heavy” isotope labeled L-Arginine U–^13^C_6_ and L-Lysine 4,4,5,5-^2^H_4_ (R_6_K_4_), or “light” labeled L-arginine and L-lysine (R_0_K_0_) (Fig. 1*A*). Trypanosome parasites have been shown to grow normally in SILAC HMI-9 and remain infective in mice (55). In order to study the cellular response to locus specific DSBs, we used two established cell lines, ^1^HR and VSG^up^, that contain the tetracycline inducible yeast I-*Sce*I homing endonuclease which induces a DSB in approximately 95% of all cells (20, 25). The ^1^HR cell line contains an 18 bp I-*Sce*I heterologous recognition sequence (*Sce*R; Fig. S1*A*) at an intergenic chromosome internal polycistronic transcription unit on one homolog of chromosome 11 (25), and the VSG^up^ strain harbors the *Sce*R upstream of the actively expressed *VSG* on BES1 on chromosome 6a (Fig. S1*B*) (19, 20). In the ^1^HR cell line approximately 60% of the cells are able to repair the DSB and survive, 85% of repair uses RAD51-dependent allelic HR and 5% ectopic HR and RAD51-independent microhomology-mediated end joining (25). In contrast, only 5% of VSG^up^ cells survive a DSB suggesting lesions at this locus are highly toxic, 60% use RAD51-dependent HR and 40% a RAD51-independent repair to resolve the DSB (20). Incorporation of labeled amino acids in the ^1^HR and VSG^up^ cell lines was assessed by mass spectrometry (MS), and we observed 93.4% and 96.1% heavy label incorporation in the ^1^HR and VSG^up^ cell lines, respectively (Fig. S2*A*). γH2A foci formation was also observed in cells grown in SILAC medium (Fig. S2*B*). These foci have been shown to form post DSB induction (20, 25) and an indicator of a robust DDR. The SILAC adapted cells lines respond to DNA damage as expected, we therefore proceeded to establishing the DNA damage phosphoproteome in *T. brucei*.

### Analysis of the total proteome following a double-strand break

In bloodstream form trypanosomes, γH2A accumulation peaks at 12 h post DSB induction in both the ^1^HR and VSG^up^ cell lines (53), and ssDNA accumulates between 9 and 12 h (20, 25). In addition, at this time point we do not see any cell death allowing us to capture phosphorylation events associated with the DDR. We therefore chose to carry out proteomic analysis at 12 h post DSB induction. For each sample, a label swap replicate was carried out (induced cells grown in “light” media and uninduced in “heavy” media). Analysis of the total peptide extract was carried out, and in the ^1^HR dataset, a total of 2457 proteins were identified (Fig. 1*B*), and only 12 of these showed a > 2-fold change in abundance following DSB induction. In the VSG^up^ proteome, a total of 2646 proteins were identified (Fig. 1*C*) only 8 of which had a > 2-fold change in abundance following DSB induction, suggesting large changes at the protein level are not seen at 12 h post DSB induction. However, we did observe a notable downregulation of the ribosomal proteins in both the ^1^HR and VSG^up^ proteomes (Fig. 1, *B* and *C*). Ribosomal proteins are important for the assembly of ribosomal subunits and also function as RNA chaperones (56). The specific downregulation of ribosomal genes observed here suggests that there may be a global inhibition of protein translation in response to DNA damage as has been reported following a CRISPR-Cas9-induced DSB in mammalian cells (57).

### The DSB locus-specific phosphoproteome of the ^1^HR and VSG^up^ strains

**Figure 2:**
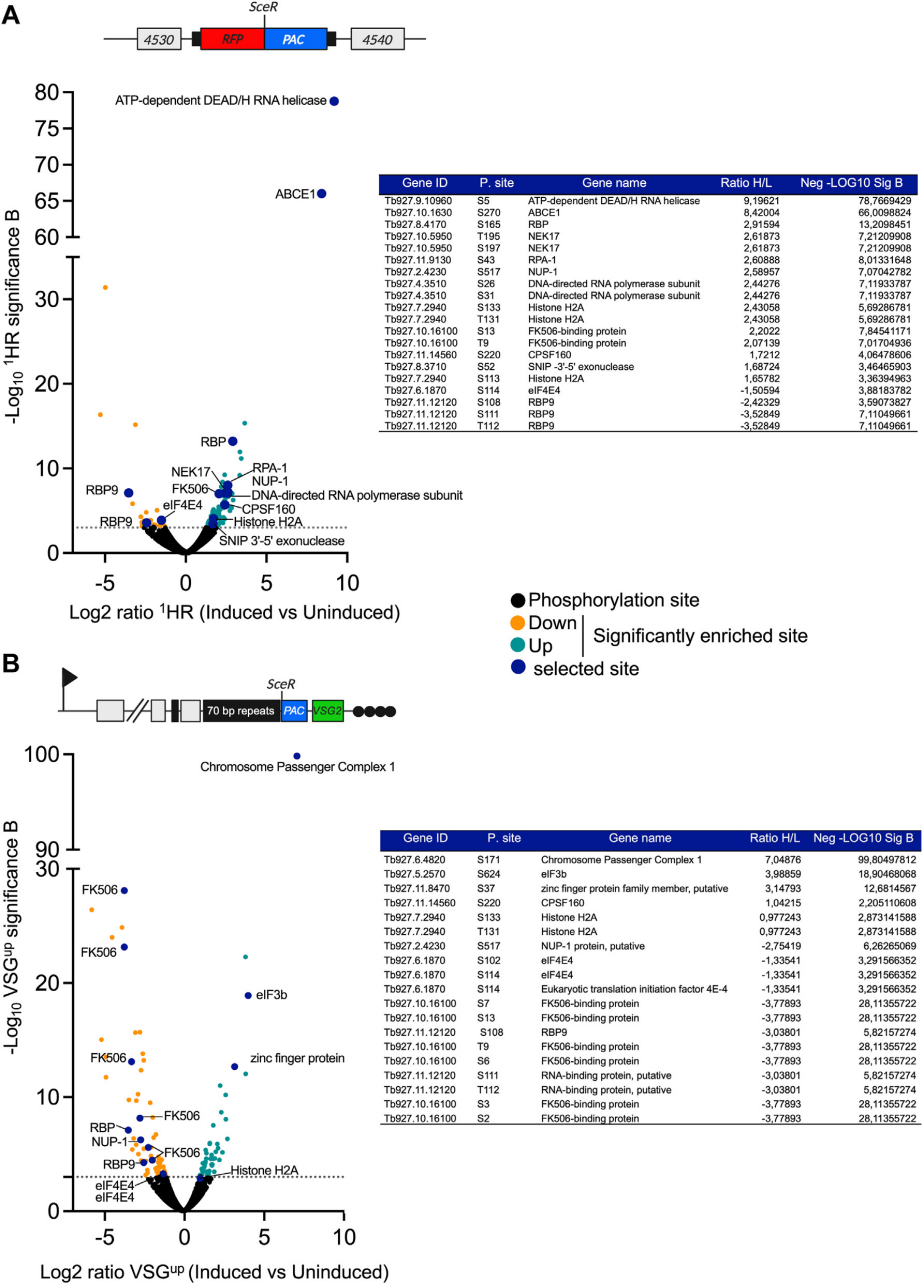
**Locus specific phosphoproteome**. *A*, *upper panel*: schematic of the ^1^HR locus. *Lower* panel: quantification of changes in phosphorylation in the ^1^HR phosphoproteome. *Black circles*, nonsignificant change in phosphorylation; enriched phosphorylation-*teal circles*, significantly increased, *orange circles* significantly decreased; *blue circles* selected proteins in table inset. *B*, *upper* panel: schematic of the VSG^up^ locus. *Lower panel*: quantification of changes in phosphorylation in the VSG^up^ phosphoproteome, details as in (*A*). HR, homologous recombination; VSG, variant surface glycoprotein.

**Figure 3:**
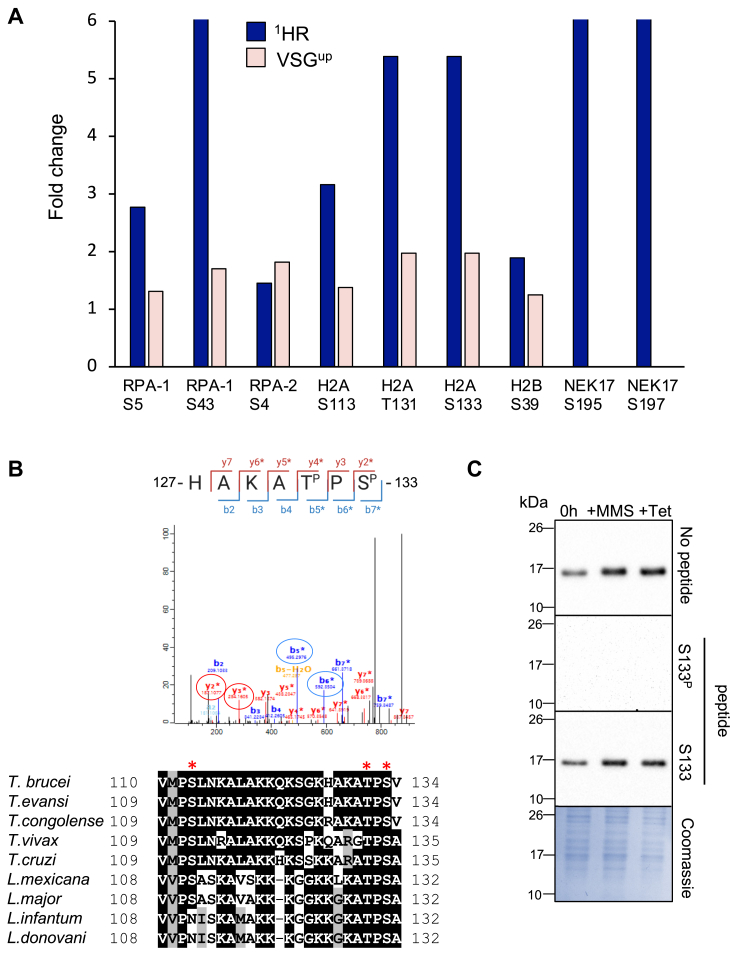
**Phosphorylation of proteins involved DNA repair and recombination.***A*, quantification of phosphorylation at specific amino acid sites. *B*, *upper panel*: Tandem mass spectral data showing assignment of T131 and S133 phosphorylation sites on H2A (Tb927.7.2940) the MaxQuant localization score for each site is 1; The loss of a phosphate group (−98 Da) is indicated with a (∗). The fragmentation pattern of the peptide is shown above. The b5 and b6 ions, highlighted on the spectrum with *blue circles*, show specific phosphorylation of T131, while the y2 and y3 ions highlighted with *red circles* show the specific phosphorylation of S133. *Lower* panel: amino acid sequence alignment of the H2A C terminus among trypanosomatids. Alignments shown are the C terminus of H2A from *Trypanosoma brucei* (Tb927.7.2940), *Trypanosoma evansi* (TevSTIB805.7.2930-t26_1), *Trypanosoma congolense* (TcIL3000_7_2140.1), *Trypanosoma vivax* (TvY486_0702710), *Trypanosoma cruzi (*TcCLB.508321.11), *Leishmania mexicana* (LmxM.08_29.1720.1), Leishmania *infantum* (LINF_210016800-T1) and *Leishmania donovani* (LdCL_210016600-t42). *Red asterisks* denote the S113, T131, and S133 phosphorylation sites; S, serine; T, threonine. *C*, Western blotting of the peptide competition assay shows the specificity of the anti-S133^P^ antibody to the phosphorylated peptide. Parasite lysates treated or nontreated with MMS/tet were separated by SDS-PAGE and probed with rat anti-S133^P^ serum, previously incubated with the phosphorylated (S133^P^) or nonphosphorylated (S133) peptides. A preincubation no peptide is included as control, and a Coomassie stain is included as a loading control for the S133^P^ blot. MMS, methyl methanesulfonate.

Within the total protein extract, phosphorylated peptides are of low abundance, and so we carried out an enrichment step using affinity purification on a TiO_2_ column (58). To identify significantly changing phosphorylation sites in the two datasets, we used significance B testing (59), which takes into account the intensity-weighted significance. Overall, there was no significant change in the distribution of phosphorylation events among phosphor serine (S) and threonine, (T) but our dataset included no phosphorylation of tyrosine (Y) (Fig. S3*A*) compared to published data. The majority of the phosphorylation events identified were on known proteins (Fig. S3*B*). In total, we identified 6905 phosphorylation sites in the ^1^HR phosphoproteome and 6540 for VSG^up^ (Figs. 2, *A* and *B*, S3, and Dataset S3). Of these sites, 5991 were common to both the ^1^HR and VSG^up^ datasets, and 914 and 549 sites were unique to the ^1^HR and VSG^up^ respectively (Fig. S3*C*) and a core set of 211 that are significantly upregulated or downregulated. Using γH2A as a positive control for the phosphoproteome analysis, in the ^1^HR strain we report an average of 5.39 fold increase in phosphorylation of γH2A at T131 (previously annotated as T130 excluding the initiator methionine (53)) (Figs. 2*A* and 3*A*) and a 1.97 fold increase in the VSG^up^ strain (Figs. 2*B* and 3*A*), confirming that we are able to detect DSB specific phosphorylation events using quantitative phosphoproteomics.

We identified 128 significantly altered phosphosites on 81 proteins in the ^1^HR phosphoproteome, 107 of which were upregulated and 22 downregulated (Fig. 2*A* and Dataset S2). In the VSG^up^ phosphoproteome, 135 significantly altered sites were identified on 95 proteins, 65 of which were upregulated and 70 downregulated (Fig. 2*B* and Dataset S2). Within the phosphoproteomes, 26 sites were significantly enriched in both the ^1^HR and VSG^up^ strains. Of these 26 phosphorylation sites, 21 were upregulated in the ^1^HR, and only 3 were upregulated in VSG^up^, with downregulation of phosphorylation making a bigger contribution to subtelomeric repair (Data S2). Among the 26 phosphosites that were significantly enriched in both the ^1^HR and VSG^up^ datasets, a number of modifications on proteins involved in RNA binding, RNA processing and translation were identified (Dataset S1). We surveyed the 211 DSB responsive phosphorylation sites for categorical enrichment of Gene Ontology (GO) terms, using a Fisher’s exact test (false discovery rate (FDR) ≤ 0.05). The significantly enriched ^1^HR and VSG^up^ phosphorylation sites were further divided into phosphoproteins that were significantly upregulated or downregulated in each dataset. In ^1^HR 6 significantly enriched GO terms were identified for upregulated phosphorylated proteins, which included histones and proteins that respond to DNA damage (Fig. S4*A*(i)), and one for VSG^up^ (Fig. S4*A*(ii)). The reverse was seen with downregulated phosphorylated proteins; RNA binding was common to both, and five terms were enriched in the VSG^up^ including chromatin organization (Fig. S4*B*(ii)) and one term enriched for ^1^HR, RNA processing (Fig. S4*B*(i)).

### Phosphorylation of DNA repair proteins in the ^1^HR and VSG^up^ strains

We next identified modifications on selected DNA repair proteins (Fig. 3*A*). In mammals, phosphorylation of histone H2B S14 on the C terminus is a late marker of DNA damage and is dependent on γH2AX (60). We detected a novel phosphorylation site at S39 on the C terminus of H2B (Tb927.10.10590) in ^1^HR strain (1.89-fold increase, *p* = 0.0339), and in VSG^up^ (1.25-fold increase, *p* = 0.38) (Fig. 3*A*). In mammalian cells, three members of the NIMA (never in mitosis gene a)-related (NEK) kinase family, NEK1, NEK1, NEK10, and NEK11 are involved in the DDR and are implicated in check point control following DNA damage (61). We saw a significant enrichment of the phosphorylation of the serine/threonine protein kinase NEK17 (Tb927.10.5950) in response to an ^1^HR DSB with two sites on NEK17, S197, and T195, increase by 6.14-fold (*p* = 6.13622 × 10^−8^) (Figs. 2*A*, 3*A* and Dataset S1). NEK17 kinase is therefore a possible candidate for implementing phosphorylation marks that are specific to chromosomal internal regions.

The heterotrimeric replication protein A (RPA) complex, consisting of RPA-1, RPA-2, and RPA-3, is the major single-strand DNA binding protein in eukaryotes, and we identified three of phosphorylation sites on this complex in our dataset (Figs. 2*A*, 3*A* and Dataset S1). In mammals and yeast, the N terminus of RPA-2 is hyperphosphorylated by the ATR kinase in response to a DNA break (2, 62) which increases the affinity of RPA-2 for RAD51 (63) and promotes repair by HR (64). We identified one phosphorylation site on RPA-2, S4, which showed a moderate upregulation of 1.45 and 1.82-fold in response to ^1^HR and VSG^up^ DSBs, respectively, suggesting that the N terminus of RPA-2 is not hyperphosphorylated at 12 h post DSB induction (Fig. 3*A*). Two sites on RPA-1 (Tb927.11.9130), the single-stranded DNA binding component of the complex (65), also had specific sites phosphorylated. The first site, S5 was previously identified in a global phosphoproteomics analysis (55), and we reported an average fold change in phosphorylation of 2.77 (*p* = 0.001) and 1.31 (*p* = 0.29) (Dataset S1) in response to DSBs induced in the ^1^HR and VSG^up^ cell lines, respectively (Fig. 3*A*). The second site, S43 showed a 6.1-fold increase (*p* = 9.69 × 10^9^) in response to an ^1^HR DSB (Fig. 3*A*), and 1.7-fold increase (*p* = 0.06) in the VSG^up^ phosphoproteome (Dataset S1). Several non-DNA repair proteins were differentially phosphorylated in response to DNA damage in our two cell lines. We identified a single phosphorylation event on S517 of NUP-1 in response to a DSB at a chromosomal internal site (Fig. 2, *A* and *B*), suggesting nuclear pore complex components may be differentially required for DSBR in *T.* brucei depending on the chromosomal context. Our data also revealed that the FK506-binding protein is phosphorylated on T9, S13 in ^1^HR but dephosphorylated on S2, S3, S6, S7, T9, and S13 (Fig. 2, *A* and *B*). On FK506, only phosphorylation of S2 has previously been identified in a global phosphoproteomics analysis (55), suggesting that de/phosphorylation of S3, S6, S7, T9, and S13 are specifically in response to DNA damage. These data suggest a number of additional proteins previously unknown to be involved in the DNA repair process in trypanosomes and could be important for repair at either a chromosome internal or subtelomeric region (Fig. 2, *A* and *B* and Dataset S1).

### Phosphorylation of S133 of histone H2A in response to DNA damage

The H2A phosphorylation on a conserved S/T-Q motif (4, 66) to give γH2AX, or γH2A in trypanosomes, is a widely studied early marker of DNA damage (53, 67). We observe robust phosphorylation of *T. brucei* γH2A (T131) in response to a DNA break (Fig. 2, *A* and *B*) and identified two additional modifications on the H2A C terminus: S113 and S133 (Fig. 3), with only T131 and S133 being conserved among trypanosomatids (Fig. 3*B*). Phosphorylation of S133 increased by 5.39-fold (*p* = 2.0283 × 10^−6^) in the ^1^HR strain and 1.97-fold (*p* = 1.3392 × 10^−3^) in the VSG^up^ strain (Fig. 3*A* and Dataset S1). Individual phosphorylation events were detected on fragments harboring exclusively T131 or S133, confirming that both sites are phosphorylated (Fig. 3*B*). A third phosphorylation site was also identified, S113, that is located on the H2A tail. The S113 site was 3.16-fold (*p* = 0.000432) upregulated in response to a DSB in the ^1^HR cell line. Residues T131 and S133 are highly conserved, but S113 shows some variation in its conservation, not present in *Leishmania infantum* or *Leishmania donovani* (Fig. 3*B*) indicating that there are lineage specific modifications to the histone tail resulting in a difference to the DDR between trypanosomatid species.

**Figure 4:**
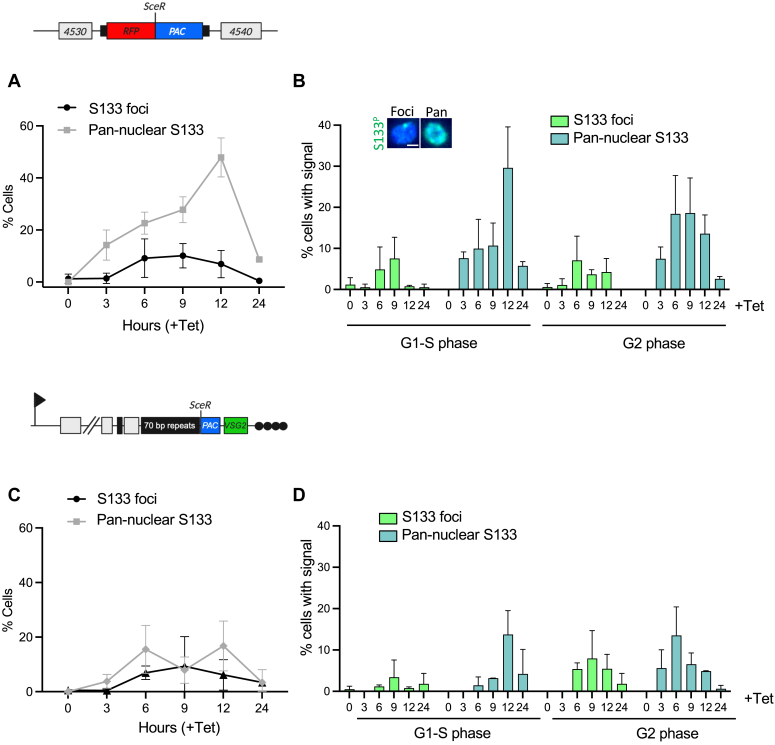
**Phosphorylation of H2A S133 following DNA damage.***Line graphs* present the general percentage of ^1^HR (*A*) or VSG^up^ (*C*) parasites showing S133 foci (*black circles*) or pan-nuclear (*gray squares*) immunofluorescent signal at 0, 3, 6-, 9-, 12-, and 24-h posttetracycline induction. Bar graphs present the percentage of ^1^HR (*B*) or VSG^up^ (*D*) cells with S133 foci (*light green bars*) or pan-nuclear (*teal bars*) signal in the G1/S or G2 cell cycle-phases post tetracycline induction. An inset image in (*B*) demonstrates the type of immunofluorescent signal displayed by the cells. The scale bar represents 1 μm. n = 100 for all times points, n = 2 technical replicates, counts performed by two independent researchers. Error bars are the standard deviation of the mean. HR, homologous recombination; VSG, variant surface glycoprotein.

To confirm that phosphorylation of H2A S133 was DNA damage responsive, we raised antibodies to the cognate phosphopeptide. We used a peptide competition assay to confirm the specificity of the antibody; here only the phosphorylated antibody was able to deplete the H2A S133 phosphorylated signal (Fig. 3*C*). We now refer to this antibody as H2A S133^P^. We next looked to see if H2A S133^P^ accumulated at the site of DNA damage in a manner similar to γH2A in trypanosomes. Using the ^1^HR and VSG^up^ cell lines, we induced DNA damage and assessed the H2A S133^P^ signal over a 24-h period. As with γH2A, we see a more robust signal in the ^1^HR cell line as compared to VSG^up^, and in both cases a higher proportion of cells showed a pan-nuclear signal, rather than single foci (Fig. 4, *A* and *C*). Cells with single foci also had a diffuse nuclear signal that was not seen in the unperturbed cells (Fig. 4*B*, inset). In the ^1^HR cell line, the 48% of cells had a pan-nuclear signal at 12 h, and 10% with single foci 9 h (Fig. 4*A*). In VSG^up^, 17% of the cells had a pan-nuclear signal and 9% had distinct foci at 12 h (Fig. 4*C*). The H2A S133^P^ signal was specific to the nucleus and characterized by a pan-nuclear signal in the ^1^HR cells line, where damage is within a megabase chromosome, while in VSG^up^ cell line, where damage is localized to the sub-telomeric regions, both pan, and subfocal accumulation was seen.

**Figure 5:**
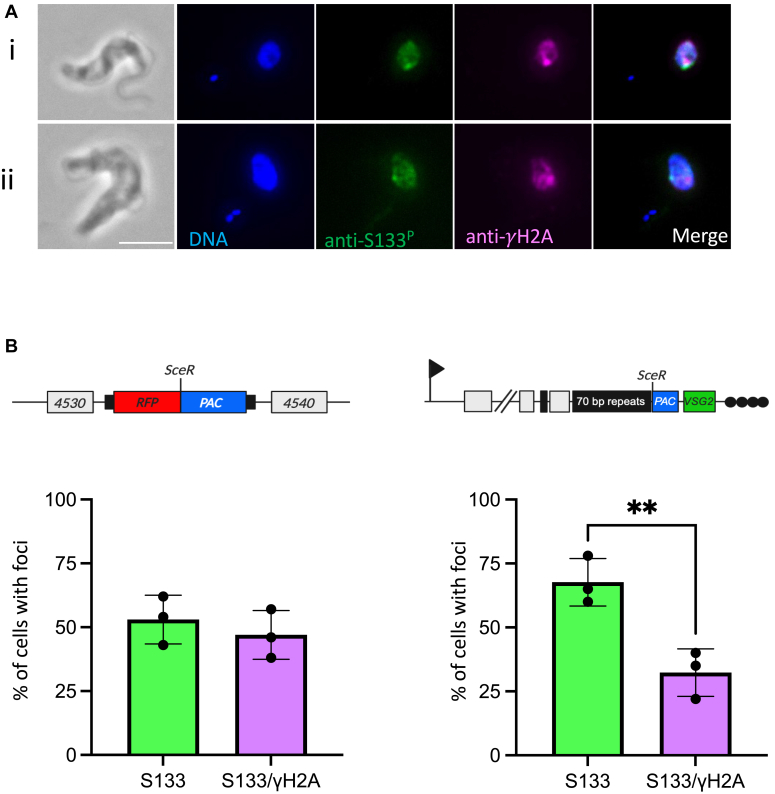
**Colocalization between H2A S133**^**P**^**and** γ**H2A.***A*, representative immunofluorescent images showing colocalization (i) or no colocalization (ii) of the anti-S133^P^ (*green*) and anti-γH2A (*magenta*) fluorescent signals (the scale bar represents 5 μm). *B*, ^1^HR and (*C*) VSG^up^ cells, the bar graphs show the percentage of cells with S133^P^ foci only (*green bars*) or colocalizing with γH2A (*magenta bars*). Each bar represents the average of three independent experiments (*black dots*). Experiments were performed in triplicate: ^1^HR, n = 230, 181, and 302 cells, and for VSG^up^, n = 180, 119, and 192 cells. A student’s *t* test showed that there is a statistically significant difference between the percentages in VSG^up^ (*p* value = 0.0096). VSG, variant surface glycoprotein; HR, homologous recombination.

Accumulation of DNA damage proteins has been shown to be cell cycle regulated, as is the case with the RAD51 recombinase and γH2A in trypanosomes (53). We therefore quantified both the pan and focal H2A S133^P^ signal at post DNA damage. Cell cycle position was defined using the nucleus and kinetoplast as cytological markers that were 4 to 6 diamidine-2-phenylindole-stained. The H2A S133^P^ signal was confined to G1/S and G2 phase cells, similar to γH2A (Fig. 4, *B* and *D*). A reduced number of those cells going through mitosis or cytokinesis showed either a pan-nuclear signal or foci (Fig. S5). The ^1^HR line showed a stronger H2A S133^P^ signal overall (Fig. 4, *A* and *B*), in line with the higher fold change seen - 5.39-fold (*p* = 2.0283 × 10^−6^) in the ^1^HR strain *versus* 1.97-fold (*p* = 1.3392 × 10^−3^) in the VSG^up^ strain (Fig. 3*A*). In both the ^1^HR and VSG^up^ cell lines, there was a stronger pan-nuclear signal in both G1-S and G2 cells (Fig. 4, *B* and *D*). Following a chromosome internal DSB, 29% of cells had a pan-nuclear signal at 12 h post DNA damage in G1-S phase cells, and in VSG^up^ at 13% at 12 and 6 h post damage in G1-S and G2 cells, respectively. We then quantified the colocalization of the γH2A and H2A S133^P^ signal in both ^1^HR and VSG^up^. For this, we selected cells with a single H2A S133^P^ focus and determined whether they coincided with γH2A (Fig. 5). In ^1^HR, 47% of the H2A S133^P^ foci colocalized with a γH2A, while only 32% did in VSG^up^. These data suggest that a nuclear-wide signal, not just focal accumulation, occurs in trypanosomes and that H2A S133^P^ accumulates at the site of DNA damage.

## Discussion

Here, we report the use of SILAC quantitative phosphoproteomics to characterize the *T. brucei* DSB phosphoproteome in response to DSBs targeted at both chromosome internal and subtelomeric loci. The phosphorylation status of a given protein is a dynamic equilibrium balanced by the actions of protein kinases and protein phosphatases. In human cells, phosphoproteomic analysis of the DDR revealed that approximately one-third of the total sites identified are dephosphorylated in response to a DNA break (9, 10), and here dephosphorylation was highly represented in VSG^up^, accounting for 51% of significantly altered modifications, indicating its important role at the subtelomeric locus. In contrast, the majority of significantly altered phosphorylation sites following an ^1^HR are upregulated, again highlighting the disparity between chromosome internal and subtelomeric repair. The dependence on phosphorylation at a chromosomal internal locus may be due in part to the dominance of repair by allelic HR at this locus, while repair at a subtelomeric expression site favors both RAD51-dependent and independent repair. While the purpose of this study was to determine the specific phosphorylation events that govern the DDR in *T. brucei*, analysis of the total proteome revealed ribosomal proteins are downregulated following a DNA break. A similar phenomenon is seen in human cells where phosphorylation of eIF2a halts translation following a DNA break *via* ribosome remodeling (57), while mouse embryonic fibroblasts exposed to global DNA damage results in transcriptional silencing in the nucleolus (68). The downregulation of ribosomal proteins identified here alludes to cross talk between the nucleolus and the DDR, as has been observed in other organisms (69). In the kinetoplastid parasite *Trypanosoma cruzi* using more wide scale DNA damage, DNA damage by ionizing radiation led to a reduction in protein translation (70) while DNA damage by gamma irradiation revealed that active translation is critical for parasite recovery (71). Interestingly, DNA damage by ultraviolet B irradiation in mammalian cells results in an overall inhibition of protein synthesis and a translational reprogramming that facilitates the specific synthesis of DDR proteins, a response that is mediated by DNA-PKcs, a DNA damage checkpoint kinase, linking the DNA damage signaling pathway with repair (72). In *T. cruzi* a similar, specific, response is seen where DNA damage by ionizing radiation associated the ubiquitin-proteosome system with DNA repair (73). These data, including the downregulation of ribosomal proteins, shown here, point to a posttranslational regulation of gene expression in response to DNA damage that may, speculatively, aid in cellular recovery. In bloodstream form trypanosomes, an I-*Sce*I generate DNA break did not lead to an increase in the expression of RAD51, rather it was suggested that a preexisting pool of RAD51 relocalizes to the site of DNA damage (25). The limited changes in total protein abundance seen here may also be a function of the damage system used—here a single DNA break *versus* more widespread damage cause by chemical or radiation damage. Analysis of the *T. brucei* heat shock phosphoproteome displayed limited changes to the proteome in comparison to the large-scale changes seen in the phosphoproteome (74)—analogous to what we report here. We suggest that larger changes in the phosphoproteome are more revealing than changes in absolute protein abundance due to phosphorylation signaling driving the DDR.

In our dataset, RPA-1 S43 was the highest upregulated phosphorylation site following a I-*SceI* break in ^1^HR, increasing by an average of 6.1-fold, suggesting that the protein is abundantly and specifically phosphorylated in response to a break. The second phosphorylation site identified on RPA-1, S5, showed a moderate increase in phosphorylation compared to that of S43. Phosphorylation of both sites was previously identified in global studies of the *T. brucei* phosphoproteome (55, 75) indicating that these modifications also play a role outside of the DDR. Histone modifications also play a key role in the DDR, regulating access to chromatin and signaling for DNA damage (6). Key to this is the phosphorylation of histone H2A at T131 to give γH2AX in trypanosomes. In our phosphoproteome, we identified two additional sites in histone H2A that are phosphorylated in response to a DNA break: S113 and S133. Given that these modifications were not previously annotated in global analysis of the *T. brucei* phosphoproteome (55, 75) they are likely specific to DSB repair. In mammals, the phosphorylation sites on the histone tail in addition to γH2AX, here S139, are involved in the mammalian response to DNA damage (66, 76), while in *Saccharomyces cerevisiae*, systematic mutation of the histone tail identified three sites that are important for efficient DNA repair (77). Antibodies raised against the phosphorylated H2A S133 indicated that both a pan-nuclear signal and subnuclear foci are formed following I-*SceI* induced damage at two distinct chromosomal loci, that predominate in G1-S and G2 phase cells. Neither type of signal persists throughout the cell cycle, declining in post mitotic cells. Colocalization with γH2A suggests phosphorylation of H2A S133 is concentrated around the site of damage but can also occur in an undamaged chromatin context. While a strong pan-nuclear signal is not seen with γH2A in *T. brucei*, it has been reported in mammalian cells (78). Although it is unclear the role the pan-nuclear γH2AX plays, it does not inhibit repair, nor does it lead to a DDR in undamaged chromatin. It remains to be seen what the pan-nuclear H2A S133^P^ function is in trypanosomes, whether there is interdependency between the two sites.

Phosphoproteomic studies of the DDR in human cells have also identified enrichment of proteins with RNA binding capacity (3) and a number of RNA binding proteins (RBPs) have been shown to have a dual role in both RNA binding and the DNA damage response. Such is the emerging evidence for the roles of RBPs in the DDR that a new class of proteins has been defined, the DNA damage response RNA binding proteins (DDRBPs) (79). Some of these DDRBPs can also bind double strand or single strand DNA and are involved in regulating R-loop formation (79, 80) by coating the nascent RNA (81, 82). R-loop formation is associated with increased DNA damage and VSG switching in *T. brucei* (23) and it is possible that some of the RBPs identified here supress R-loop formation following DSB induction. However, it is of note that in yeast R-loop formation is an important part of efficient HR (83, 84), and therefore possible that RBPs assist in productive R-loop formation that contributes to specifically double stand break repair in trypanosomes.

Although not DNA repair proteins, both NUP-1 and FK506-binding protein (Fig. 2, *A* and *B*) have been shown to be directly involved in DNA repair and recombination in yeast and mammalian cells, respectively. Several components of the yeast nuclear pore complex are phosphorylated in response to DNA damage, including NUP-1 at S637 (11), and have been shown to associate with damaged DNA and influence repair. In *T. brucei*, NUP-1 (Tb927.2.4230; nucleoporin 1) is organized into a lattice-like network at the nuclear periphery and maintains both nuclear architecture and chromatin organization (85). In our dataset, the phosphorylation status of NUP-1 following DNA damage is chromosome context dependent: It is phosphorylated in ^1^HR and dephosphorylated in VSG^up^ cells, perhaps playing a role in repair pathway choice. FK506-binding proteins alter protein conformation through cis-trans isomerization of prolyl-peptide bonds, and in mammalian cells directs repair by promoting HR, potentially through its catalytic activity remodeling the chromatin environment (86). In trypanosomes, multiple sites in the FK506-binding protein are differentially phosphorylated, and again this is dependent on the chromatin context—being phosphorylated in ^1^HR and dephosphorylated in VSG^up^ cells.

Protein phosphorylation is a dynamic process (87) and our results show only a snapshot of the DDR capturing the response at 12 h post DSB induction. Other post translational modifications are also important to both the DDR (6, 88, 89) and *VSG* switching, with histone methylation and acetylation important for antigenic variation (41, 90, 91), and SUMOylated proteins enriched at the active BES (92). This study is the first DNA damage phosphoproteome in *T. brucei,* and we have identified an abundance of novel proteins involved in the DDR. We have also revealed an overall trend toward phosphorylation following a break in a chromosomal internal region (^1^HR) and dephosphorylation following a break at a subtelomeric expression site (VSG^up^). We speculate this may reduce the stringency of DNA repair *via* HR which may be advantageous for antigenic variation. This remains to be tested. Validation of candidate phosphorylation sites from this dataset will provide key insights into the protein modifications that govern both DSBR and antigenic variation in *T. brucei.*

## Experimental procedures

### Trypanosome strains and culturing

*T. brucei* Lister 427 cell lines were grown in HMI-11 medium at 37.4 °C (75) with 5% CO_2_ and the density of cell cultures measured using a hemocytometer. The VSG^up^ cell line has been described previously (20) and the ^1^HR cell line in (25).

### HMI-9 SILAC medium

For all proteomic analysis, HMI-9 SILAC medium (55) minus L-Arginine and minus L-Lysine (Gibco, reference 074–91211A) was used. A 17.91 g pot of powder medium was used to make up 1L of medium by the addition of 900 ml H_2_O, 2 g sodium bicarbonate (Sigma-Aldrich), and 14 μl of beta-mercaptoethanol, and the mixture stirred for 1 h at room temperature. The pH was adjusted to 7.3, and the medium filtered using a 0.2 μM filter. The following components were added to the filtered medium: 10 ml of GlutaMAX (Thermo Fisher Scientific), 100 ml dialyzed SILAC fetal bovine serum (FBS) (3 kda molecular weight cutoff) (DC Biosciences) Gibco, 5 ml Pen/strep (5000 U/ml penicillin and 5000 μg/ml streptomycin) and heavy or light labeled L-arginine and L-lysine to the concentrations stated in Table 1 to make heavy and light medium, respectively. The final concentration of L-arginine is 120 μM and L-Lysine 240 μM.

**Table 1:** The concentration of light and heavy labeled L-Arginine and L-Lysine used to supplement both IMDM and HMI-9 SILAC medium

Amino acid	Label	Source	Mw	Final concentration
L-Arginine.HCl (R0)	Light	Sigma	210.6	25.8 mg/l
L-Arginine.HCl-U-^13^C_6_ (R6)	Heavy	CIL	216.6	25.9 mg/l
L-Lysine.HCl (K0)	Light	Sigma	182.6	43.8 mg/l
L-Lysine.2HCl-4,4,5,5-^2^H_4_ (K4)	Heavy	CIL	223.1	53.5 mg/l

CIL – Cambridge Isotope Labs, UK.

### Assessing incorporation of the stable isotope label

To assess incorporation of the isotope label, the ^1^HR and VSG^up^ cell lines were seeded in “heavy”, and “light” labeled SILAC HMI-9 and after 7 days 1 × 10^8^ cells harvested from each cell culture. Samples were extracted by filter aided sample preparation (FASP) and processed for MS as described below.

### Phosphoproteomic experimental set up

For preparation of samples for proteomic and phosphoproteomic analysis, ^1^HR and VSG^up^ cell lines, grown in SILAC media for 7 days as detailed in the section above, were seeded in SILAC “heavy” and “light” medium and cells grown in “heavy” medium were induced using 1 μg/ml of tetracycline for 12 h. For each experimental condition, a label swap replicate was carried (induced cells grown in “light” media and uninduced in “heavy” media; Dataset S3). Approximately 3 × 10^8^ cells were harvested from each culture condition by centrifugation at 1000*g* for 10 min at 4 °C. The supernatant was removed, and the cell pellet resuspended in 200 μl ice cold PBS and transferred to a microcentrifuge tube, where it was centrifuged at 12,000*g* for 15s and the supernatant discarded. The cell pellet was lysed at 0.5 × 10^9^ cells/ml in ice-cold lysis buffer (0.1 mM N-tosyl-L-lysine-chloromethyl ketone, 1 μg/ml leupeptin, 1× phosphatase inhibitor cocktail II tablet (Calbiochem), 1 mM PMSF, and 1 mM benzamidine) and incubated at room temperature for 5 min, and the cell lysis was verified by microscopy. Cell lysates were then stored at −80 °C before further processing.

### FASP protocol

The preparation of peptides for MS analysis was carried out using FASP (93) that has been optimized for *T. brucei* (55). For the four samples generated for investigation of the DSB response, the total amount of protein concentrate was 1 mg and 2 mg of protein for each of the ^1^HR label swap replicates, and 0.89 and 0.62 mg of protein for each of the VSG^up^ replicates. For digestion of peptides, the concentrated sample was removed from the ultracentrifugal filter and a 1:50 ratio of mass spectrometry grade Trypsin Gold (Promega) added to the sample which was incubated with shaking for > 12 h at 37 °C in a thermal heat block. Trypsin digestion was inhibited by adding 0.1% formic acid (FA) to the digest.

### Peptide desalting

Peptides were desalted using a Sep-Pak C18 SPE 360 mg cartridge (Waters) using a vacuum manifold according to manufacturer instructions. All buffers were freshly prepared. Briefly, C18 phase (Sep-Pak, Waters) was activated in methanol, rinsed once in 80% acetonitrile (ACN) with 0.1% FA, washed thrice in 0.1% FA. The sample was then loaded onto the cartridge twice. Resin was washed thrice in 0.1% FA and peptides were eluted in 50% ACN with 0.1% FA. The resulting sample was dried in a SpeedVac vacuum concentrator (Thermo Fisher Scientific) until 50 μl remained and then transferred to a nano LC tube (Thermo Fisher Scientific) and dried by lyophilization.

### Phosphopeptide enrichment

Phosphopeptide enrichment and MS was carried out at the Mass Spectrometry for Biology Utechs (MSBio) platform at Institut Pasteur. Phosphopeptide enrichment was performed using a GELoader spin tip using EmporeTM C8 (3M) prepared for StageTip (Rappsilber et al.2007) and washed sequentially with 100% MeOH and 30% ACN, 0.1% trifluoroacetic acid (TFA). Before the enrichment step, 10 mg/ml TiO_2_ slurry (Sachtopore-NP TiO_2_, 5 μm, 300 Å, Sachtleben) was prepared in 30% ACN, 0.1% TFA and introduced into the GELoader C8 spin tip. The spin column was packed by centrifugation at 100*g* and then equilibrated loading buffer (80% ACN, 6% TFA, 1 M glycolic acid) before loading lyophilized tryptic peptides resuspended loading buffer at a ratio of 1:5 peptides to beads. An aliquot (10 μg) of tryptic peptides was retained for proteome analysis. TiO_2_ spin tip was first washed with 80% ACN, 6% TFA, and then with 50% ACN, 0.1% TFA at 200*g*. Phosphopeptides were eluted from TiO_2_ beads by transfer to into a new microcentrifuge tube containing 20% FA, using 10% NH_4_OH solution *via* centrifugation at 100*g*. To prevent the loss of phosphopeptides retained by the C8 plug a second elution was carried out with 80% ACN, 2% FA *via* centrifugation at 100*g*. Eluate fractions were combined and lyophilized prior to mass spectrometry analysis.

### Mass spectrometry analysis

Peptides were analyzed on a Q-Exactive HF instrument (Thermo Fisher Scientific) coupled with an EASY nLC 1200 chromatography system (Thermo Fisher Scientific). Samples were loaded at 900 bars on an in-house packed 50 cm nano-HPLC column (75 μm inner diameter) with C18 resin (3 μm particles, 100 Å pore size, Reprosil-Pur Basic C18-HD resin) and equilibrated in 98% solvent A (H_2_O, 0.1% FA) and 2% solvent B (ACN, 0.1% FA). For both proteome and phosphoproteome analysis, peptides were eluted using a three to 29% gradient of solvent B during 105 min, then a 29 to 56% gradient of solvent B during 20 min and finally a 56 to 90% gradient of solvent B during 5 min all at 250 nl/minute flow rate. The instrument method for the Q-Exactive HF was set up in the data dependent acquisition mode. After a survey scan in the Orbitrap (resolution 60,000), the 12 most intense precursor ions were selected for higher energy collisional dissociation fragmentation with a normalized collision energy set up to 26. Precursors were selected with a window of 2.0 Th. Tandem MS spectra were recorded with a resolution of 15,000. Charge state screening was enabled, and precursors with unknown charge state or a charge state of 1 and > 7 were excluded. Dynamic exclusion was enabled for 30 s. For phosphoproteomic analysis, technical replicates were carried out in which samples acquisition was repeated twice for each label swap replicate.

### MS data processing

All raw data were searched using MaxQuant software version 1.6.10.43 (https://www.maxquant.org/) (59, 94), which incorporates the Andromeda search engine (95), against the *T. brucei* 927 genome downloaded from TritrypDB (http://www.tritrypdb.org/) (Version 47, 11,074 protein sequences) (96) supplemented with frequently observed contaminants (such as mammalian keratins, porcine trypsin, and bovine serum albumins (BSAs)). All SILAC features were selected by default using the appropriate heavy K and R amino acid to be detected. Modifications included carbamidomethylation (Cys, fixed), oxidation (Met, variable) and *N-*terminal acetylation (variable) and *N*-pyroglutamate (variable) and phosphorylation (S, T, and Y variable). The mass tolerance was set to 6 parts per million (ppm) and peptides were required to be minimum 7 amino acids in length. Matching between runs allows peptides that are present in one sample but not identified by tandem MS in all samples to be identified by similarities in retention times and mass. The FDRs of 0.01 was calculated from the number of hits against a reversed sequence database. Only phosphorylation sites with a MaxQuant localization probability > 0.95 were considered.

### Statistical analysis of proteomic data

Statistical analysis was carried out using Perseus (94) version1.6.1.3 (https://maxquant.net/perseus/). SILAC ratios were transformed to Log_2_ and intensities to Log_10_. Values were subject to further quality filtering such that ratios with >100% variation between label swap replicates were removed, and the localization probability of each phosphorylation site was required to be ≥ 0.95. Significantly changing phosphorylation sites were identified using significance B testing (59) which takes into account the intensity-weighted significance and used a Benjamini-Hochberg correction (97) to set the FDR at ≤ 0.01. Categorical enrichment was calculated using a Fisher’s exact test with an FDR ≤ 0.01. GO term enrichment was carried out on the proteins with significantly enriched phosphosites for the ^1^HR and VSG^up^ datasets, using a Fisher’s exact test (FDR ≤ 0.05). All other statistical analysis was carried out in Microsoft Excel and GraphPad Prism, version 10 (https://www.graphpad.com).

### Anti-S133^P^ antibody

Anti-S133^P^ antisera was raised in rats using a keyhole limpet hemocyanin-conjugated phosphopeptide, C-KSGKHAKATP[pS]V (Davids Biotechnologie GmbH). Antiserum was affinity purified using the corresponding peptides.

### Peptide competition assay

For the peptide competition assay, primary antibody Anti-S133^P^ was preincubated with 40 ng ml^−1^ of the phosphopeptide KSGKHAKATP[pS]V, the peptide KSGKHAKATPSV or the equivalent volume in water, in PBS 0.01% Tween 20 with 3% BSA, for 1 h at room temperature prior to incubation with the immunoblot.

### Immunoblotting

Approximately 1 × 10^7^ parasites were treated with 1 μg mL^-1^ tetracycline for 12 h, or 0.0003% methyl methanesulfonate for 24 h. Western blotting was carried out according to standard protocols, except that 1× phosphatases inhibitor PhosSTOP (Roche) was added to the lysis buffer, and samples were separated on a 15% SDS-PAGE gel. Immunoblots were blocked in PBS 0.01% Tween 20 with 5% BSA. Primary *T. brucei* Anti-S133^P^ antibody was used at a 1:250 dilution and secondary goat anti-rat IgG horseradish peroxidase (Bio-Rad) was used at a 1:10,000 dilution. Blots were revealed by chemiluminescence using the Amersham ECL Prime Western Blotting Detection Reagent Kit (GE HealthCare) and a ChemiDoc Touch Gel Imaging System.

### Immunofluorescence analysis

Immunofluorescence was carried out according to standard protocols. In brief, cells were fixed in final 1% volume (v/v) formaldehyde, on ice for 30 min. Fixed cells were centrifuged for 1 min at 1000*g* and washed with 1 ml ice cold PBS, twice. Cells were settled onto poly-l-lysine treated slides for up to 30 min and washed 3 × 5 min in PBS. Blocking was carried out for 15 min in 50% FBS in PBS, and all antibody dilutions were in 3% FBS. Primary antibodies rat anti-S133^P^ and rabbit anti-γH2A (53) were used at a concentration of 1:250. Secondary antibody, goat anti-rat AF488 (AlexaFluor plus, Invitrogen, Lot #XI350194) and goat anti-rabbit AF555 (AlexaFluor plus, Invitrogen, Lot #VC297826) was used at a concentration of 1:1000. Cells were mounted in Vectashield (Vectorlabs) containing 4 to 6 diamidine-2-phenylindole. Images were acquired using a ZEISS Axio Imager Z2 epifluorescence microscope combined with an Axiocam 506 mono camera. Acquisition software Zen 2.3 (blue edition) (https://www.zeiss.com), version 2.3.69.1005. Images were processed using Image J2, version 2.14/1.54f (98) (https://imagej.net/software/fiji/downloads). Statistical analysis was carried out in GraphPad Prism, Version 10 (https://www.graphpad.com).

## Data availability

The mass spectrometry proteomics data have been deposited to the ProteomeXchange Consortium *via* the PRIDE (99) partner repository with the dataset identifier PXD034455.

## Supporting information

This article contains supporting information.

## Conflict of interest

The authors declare that they have no conflicts of interest with the contents of this article.
