# Supplementary figures and images for: Phosphoproteomic analysis of the response to DNA damage in Trypanosoma brucei
Source: J Biol Chem. 2024 Aug 14;300(9):107657. doi: 10.1016/j.jbc.2024.107657 (PMC11408851; doi:10.1016/j.jbc.2024.107657)

Figure S1

A

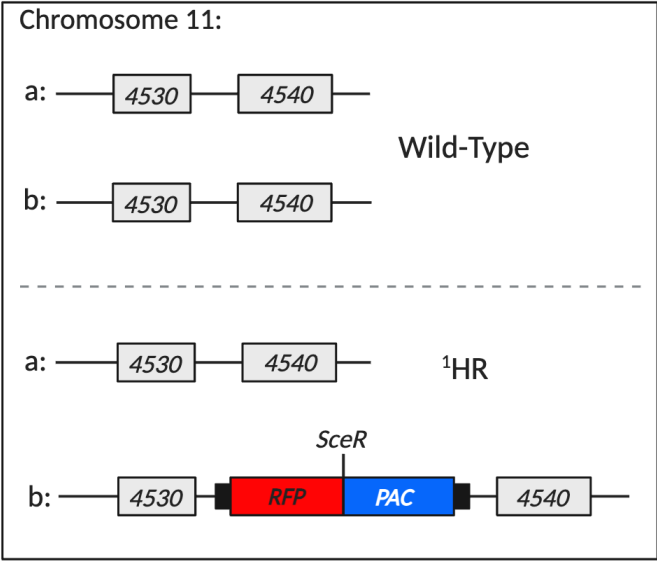

B

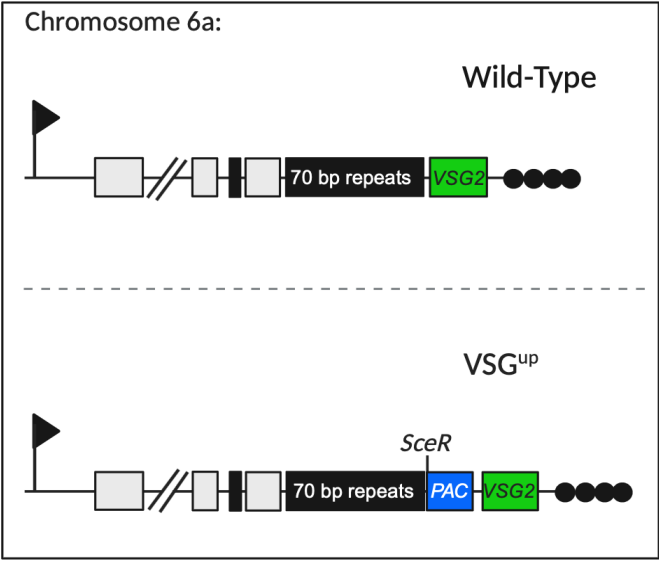

Supplement: Figure S1 [file mmc1.pdf]

Figure S3.

A

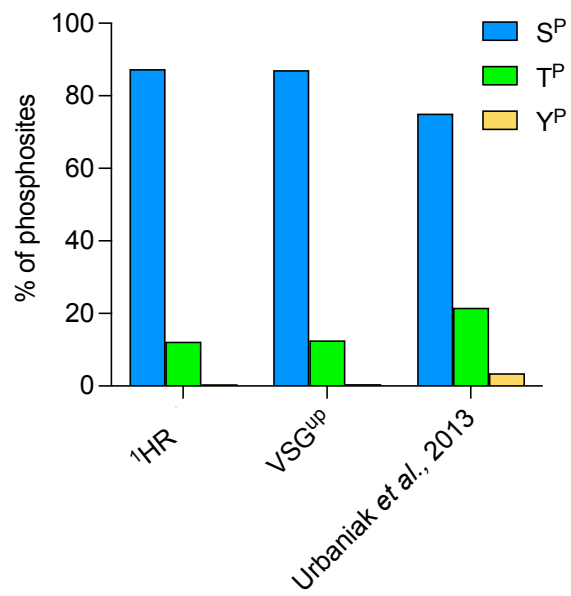

B

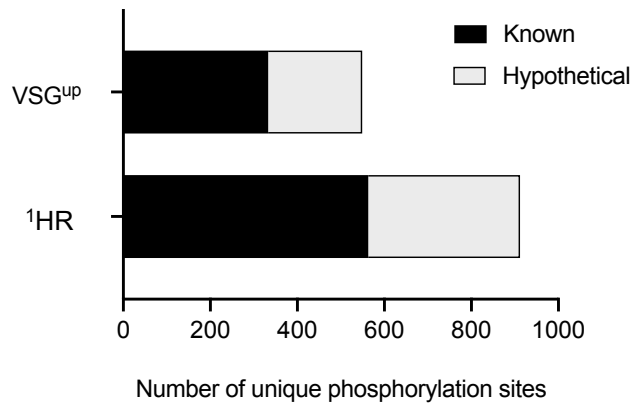

C

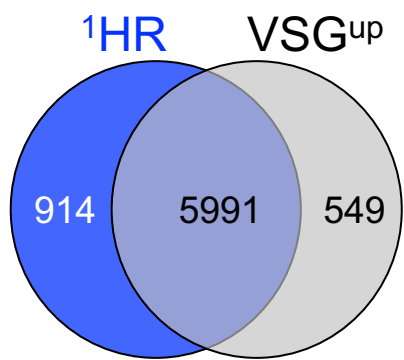

Supplement: Figure S3 [file mmc3.pdf]

Figure S4.

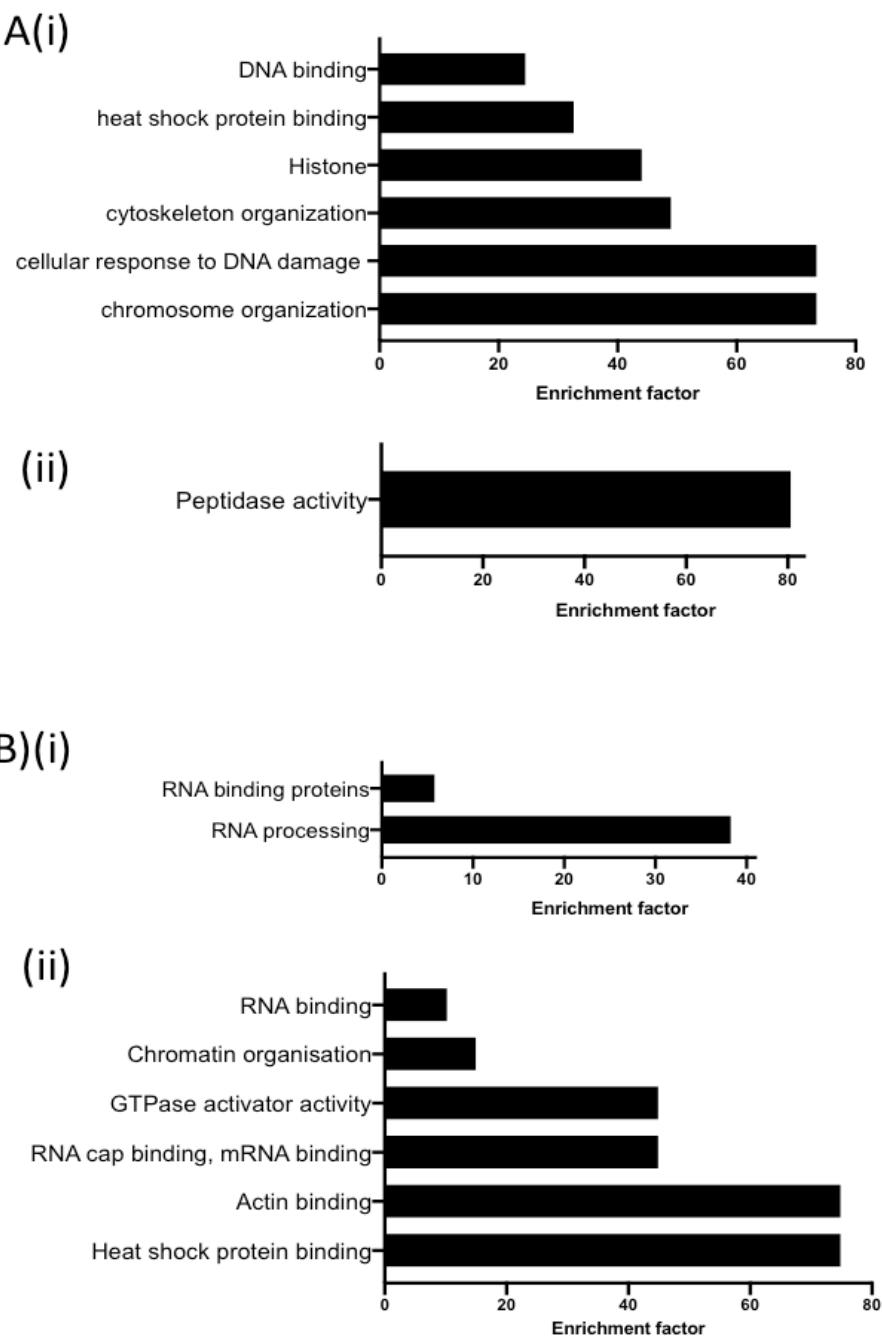

Supplement: Figure S4 [file mmc4.pdf]

Figure S5.

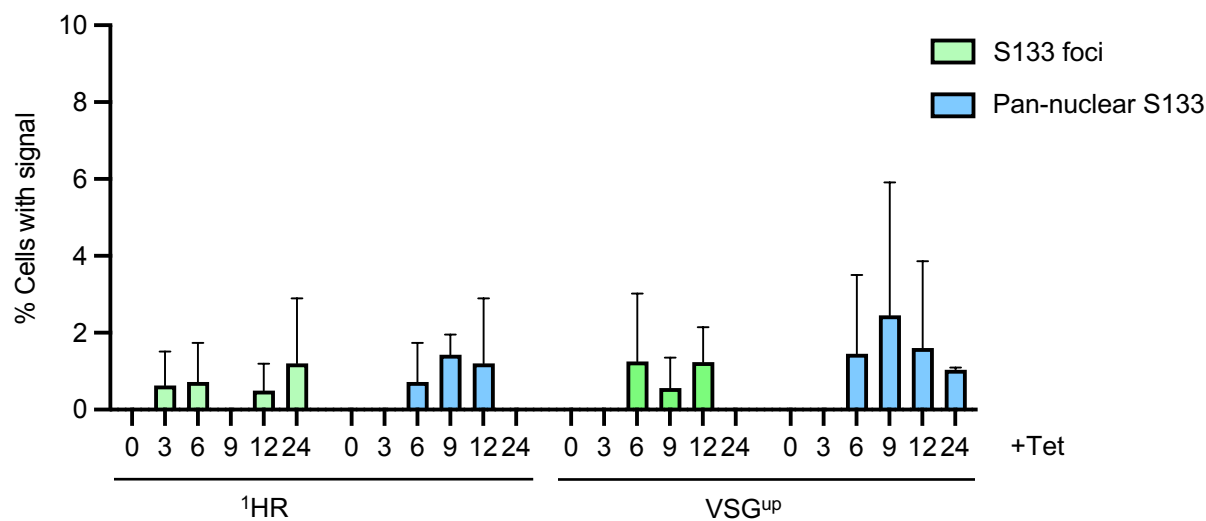

Supplement: Figure S5 [file mmc5.pdf]
