# Supplementary material for: Phosphoproteomic analysis of the response to DNA damage in Trypanosoma brucei
Source: J Biol Chem. 2024 Aug 14;300(9):107657. doi: 10.1016/j.jbc.2024.107657 (PMC11408851; doi:10.1016/j.jbc.2024.107657)

Figure S2

A

Cell line	Number of peptides	Number of 'heavy' labelled peptides	Incorporation
^{1}HR	12, 006	11, 217	93,3%
VSG^{up}	17, 163	16, 500	96,1%

B

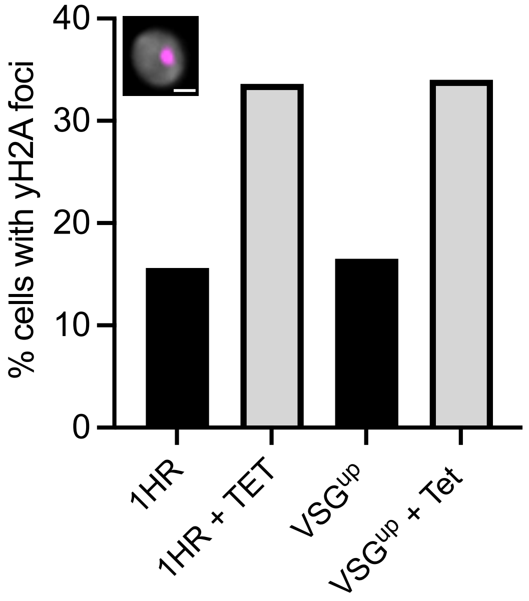

Supplement: Figure S2 [file mmc2.pdf]
