# Supplementary material for: Phosphoproteomic analysis of the response to DNA damage in Trypanosoma brucei
Source: J Biol Chem. 2024 Aug 14;300(9):107657. doi: 10.1016/j.jbc.2024.107657 (PMC11408851; doi:10.1016/j.jbc.2024.107657)
Supplement: Supporting information [file mmc9.docx]

**Supporting information (for publication)**

**Supporting Figure 1. The ^1^HR and VSGup cell lines** (A) Schematic of chromosome 11 Tb927.11.4530/40 locus, showing both alleles (upper panel: wild type) and then following modification to generate the ^1^HR chromosome-internal DSB cell line with the I-*Sce*I recognition site, SceR, highlighted (lower panel: ^1^HR). The DSB site is flanked upstream by *red fluorescent protein (RFP)* and *puromycin-N-acetyltransferase (PAC)* downstream. The site is positioned at an intergenic region between Tb927.11.4530 and Tb927.11.4540, shown as ‘4530’ and ‘4540’, respectively. Black boxes are tubulin intergenic sequences. VSG^up^ cell line set up showing the modified BES1 on chromosome 6a. An I-*SceI* meganuclease recognition site is inserted upstream of the actively expressed *VSG-2*, shown with a red vertical line. The *SceR* is flanked downstream by a *puromycin-N-acetyltransferase* gene (PAC). Arrow; native promoter of the expression site, white boxes; genes, solid black box; 70 bp repetitive sequence, black circles; telomere.

**Supporting Figure 2. Adaptation of the ^1^HR and VSGup cell lines to growth in SILAC medium.** (A) Incorporation of heavy and light labels. (B) γH2A foci formation in cell lines adapted to growth in SILAC HMI-9 medium. ‘- Tet’ indicates uninduced cells, ‘+ Tet’ indicates 12 h DSB induction. n > 100 for each count. Inset, example of a γH2A positive nuclei, with γH2A shown in magenta and the DAPI nuclear stain in grey. Scale bar 1 µm.

**Supporting Figure 3. *T. brucei* DNA damage phosphoproteome.** (A) Distribution of the total phosphorylation sites identified in the ^1^HR and VSG^up^ DNA damage phosphoproteomes amongst phospho serine (S^P^), threonine (T^P^) and typrosine (Y^P^). A comparison to the published global phosphoproteome of *T. brucei* is shown (54). (B) Number of phosphorylation sites in the ^1^HR and VSG^up^ phosphoproteomes that are located on proteins identified as hypothetical or hypothetical conserved. (C) Venn diagram of the total number of phosphosites observed in ^1^HR and VSG^up^ phosphoproteomes, with those sites identified in both data sets indicated.

**Supporting Figure 4. Categorical enrichment of GO terms DNA damage responsive phosphoproteins. (**A) GO terms enriched in proteins whose phosphorylation increases following (i) an ^1^HR DSB and (ii) a VSG^up^ DSB. (B) GO terms enriched in proteins whose phosphorylation decreases following (i) an ^1^HR DSB and (ii) a VSG^up^ DSB.

**Supporting Figure 5.** Percentage of ^1^HR or VSG^up^ cells with S133 foci (light green bars) or pan-nuclear (blue bars) immunofluorescent signal in the post-mitotic cell cycle-phase at 0, 3, 6-, 9-, 12- and 24-hours post-tetracycline induction. n=100 for all times points, n=2 technical replicates, counts performed by two independent researchers. Error bars are the standard deviation of the mean.

**Supporting dataset 1.** Significantly changing phosphosites in ^1^HR and VSG^up^.

**Supporting dataset 2.** Significantly enriched phosphosites in ^1^HR and VSG^up^.

**Supporting dataset 3.** Technical replicates and label swap for ^1^HR and VSG^up^.
